# LncRNA ST8SIA6-AS1 promotes hepatocellular carcinoma cell proliferation and resistance to apoptosis by targeting miR-4656/HDAC11 axis

**DOI:** 10.1186/s12935-020-01325-5

**Published:** 2020-06-11

**Authors:** Qiang Fei, Feihong Song, Xinwei Jiang, Han Hong, Xiaoyong Xu, Zhengkang Jin, Xiang Zhu, Binghua Dai, Jiamei Yang, Chengjun Sui, Minhui Xu

**Affiliations:** 1 Department of Hepato-Pancreato-Biliary Surgery, The Affiliated Suzhou Hospital of Nanjing Medical University, 215001 Suzhou, China; 2 Department of Special Treatment and Liver Transplantation, Shanghai Eastern Hepatobiliary Surgery Hospital, Shanghai, 200438 China

**Keywords:** ST8SIA6-AS1, HDAC11, miR-4656, Hepatocellular carcinoma cell lines, Cell proliferation, Apoptosis

## Abstract

**Background:**

Dysregulation of long non-coding RNAs (lncRNAs) results in development of human diseases including hepatocellular carcinoma (HCC). Although several HCC related lncRNAs have been reported, the biological functions of many lncRNAs during the development of HCC remains unknown.

**Methods:**

The expression of ST8SIA6-AS1 was studied by realtime PCR (RT-qPCR) and bioinformatic analysis. The biological functions of ST8SIA6-AS1 was examined by CCK-8 assay and flow cytometry analysis. The target of ST8SIA6-AS1 was analyzed by bioinformatic analysis and validated by dual luciferase reporter assay, western blotting and RT-qPCR.

**Results:**

In this study we demonstrated that ST8SIA6-AS1 was an upregulated lncRNA in hepatocellular carcinoma. SiRNA-mediated knockdown of ST8SIA6-AS1 repressed cell proliferation and induced cell apoptosis in HCC cells. Bioinformatic analysis and RT-qPCR further showed that ST8SIA6-AS1 mainly located in cytoplasm. Dual luciferase reporter assay further revealed that ST8SIA6-AS1 interacted with miR-4656 in HCC cells. In addition, HDAC11 was identified as a target gene in HCC cells and ST8SIA6-AS1 could upregulate HDAC11 via sponging miR-4656. Transfection of recombinant HDAC11 partially rescued the inhibition of cell proliferation and increase of cell apoptosis inducing by knockdown of ST8SIA6-AS1.

**Conclusion:**

In conclusion, our findings suggested that ST8SIA6-AS1 was a novel upregulated lncRNA in HCC and could facilitate cell proliferation and resistance to cell apoptosis via sponging miR-4656 and elevation of HDAC11, which might be a promising biomarker for patients with HCC.

## Background

According to statistics, liver cancer is the sixth most commonly diagnosed cancer type globally in 2018 [1]. Liver cancer is a relative lethal cancer type, accounting for 8.2% of cancer-related deaths [1]. Hepatocellular carcinoma (HCC) is the major type of liver cancer, which represent about 90% of cases [2]. For patients with advanced HCC, the conventional chemotherapy demonstrated no survival advantage and currently used targeted therapy agent showed relatively low response rate [3]. Hence, investigation of molecular mechanisms of HCC is imperative to provide novel targets for treatment of HCC.

Long non-coding RNAs (lncRNAs) are 200 nucleotides in length molecules with no protein coding potential [4]. According to well-characterized competing endogenous RNA (ceRNA) hypothesis, lncRNA can sponge microRNAs (miRNAs) via complementary sequences and upregulates expression of miRNA target genes [5]. Due to the critical roles of miRNAs in cancer progression, lncRNAs are also involved in carcinogenesis [6, 7]. In HCC, dysregulation of lncRNAs contributed to cancer cell proliferation and resistance to cell apoptosis. For example, lncRNA MCM3AP-AS1 promoted cell proliferation and cell cycle progression in HCC cells via sponging miR-194-5p and upregulation of FOXA1 [8]. LncRNA profiling in HER2 + breast cancer firstly identified ST8SIA6-AS1 as a cancer-associated lncRNA [9]. Experimental analysis showed that ST8SIA6-AS1 regulated cell proliferation, migration and apoptosis in breast cancer cells [10]. The expression and function of ST8SIA6-AS1 was not known.

Histone deacetylases (HDACs) play important roles in physiological processes via removal of acetyl groups from histone and other proteins [11]. Studies indicated that HDACs were implicated in cancer cell proliferation, metastasis, resistance to apoptotic signal and drug resistance [12–14]. Overexpression of HDACs were found in several cancer types [15]. In HCC, RT-qPCR and western blotting results showed that HDAC11 was the only upregulated HDAC member [16]. Inhibition of HDAC11 led to p53-dependent cell apoptosis in HCC cells [16]. However, it remains unknown how HDAC11 was elevated in HCC.

In the present study, our analysis of previous data showed that ST8SIA6-AS1 was one of most significantly upregulated lncRNAs in HCC. We aimed to study the biological function of ST8SIA6-AS1 in HCC and revealed the molecular mechanisms of ST8SIA6-AS1 in HCC cells.

## Materials and methods

### Patient samples

70 patients with HCC were treated with surgery to remove the tumors and matched normal tissues in Shanghai Eastern Hepatobiliary Surgery Hospital during July 2013 to September 2017. The inclusion criteria were as follows: clear imaging, complete patient information and pathological diagnosis. The exclusion criteria were as follows: no previous chemotherapy or radiotherapy before surgery. All patients provided written informed consents before the enrollment. No patient received chemotherapy or radiotherapy before the surgery. The protocol of this study was approved by the Ethical Committee of Shanghai Eastern Hepatobiliary Surgery Hospital (Approval number: EHSH20130703). The tissues were stored in −80 °C refrigerator before subjected to RNA extraction.

### Cell culture

The immortalized human liver cell line (THLE-2) and HCC cell lines (Huh7, MHCC97 and Hep3B) were bought from American Type Culture Collection (Manassas, VA). Cells were cultured with DMEM (Invitrogen; Thermo Fisher Scientific, Waltham, MA) supplemented with 10% FBS (Hyclone, Logan, UT) 100 U/ml penicillin (Invitrogen; Thermo Fisher Scientific), 0.1 mg/ml streptomycin (Invitrogen; Thermo Fisher Scientific). The cells were maintained in a humid incubator with 5% CO_2_ at 37 °C.

### siRNA-mediated gene knockdown and plasmid transfection

ST8SIA6-AS1 siRNA-1, ST8SIA6-AS1 siRNA-2 and control siRNA were synthesized by GenePharma (Suzhou, China). ST8SIA6-AS1 siRNA-1, ST8SIA6-AS1 siRNA-2 or control siRNA was transfected into cells with Lipofectamine 3000 reagent (Invitrogen; Thermo Fisher Scientific) following manufacturer’s protocol. Full length of ST8SIA6-AS1 and HDAC11 coding sequence (CDS) was PCR amplified from Huh7 cDNA and ligated into pcDNA3. pcDNA3 or pcDNA3-ST8SIA6-AS1 or pcDNA3-HDAC11 was transfected into cells with Lipofectamine 3000 reagent following manufacturer’s protocol.

### Overexpression of miR-4656

miR-4656 mimic and miR-NC were synthesized in GenePharma. miR-4656 mimic or miR-NC was transfected into cells with Lipofectamine 3000 reagent following manufacturer’s protocol.

### Realtime PCR

The total RNA was extracted from tissues and cells with TRIzol reagent (Invitrogen; Thermo Fisher Scientific) following manufacturer’s protocol. RNA was reverse transcribed into first-stranded cDNA with M-MLV Reverse Transcriptase (Invitrogen; Thermo Fisher Scientific). The realtime PCR was performed with SYBR Green Master Mix (TaKaRa, Tokyo, Japan) on an ABI PRISM 7300 Sequence Detection system (Applied Biosystems, Foster City, CA). The 2^−ΔΔCt^ method was used to calculate the relative mRNA/lncRNA or miRNA expression via normalization to β-actin or U6. The primer sequences were listed in Table 1.

**Table 1:** Primer sequences

Primer	Sequence
ST8SIA6	
Forward	5′-TCCTGATTCAGTGGCATGGT-3′
Reverse	5′-AGGGTTTCTTCGGTCGTCAT-3′
HDAC11	
Forward	5′- GGGTGCCCATCCTTATGGTG-3′
Reverse	5′- CAGCGGTGTGTCTGAGTTCT-3′
β-actin	
Forward	5′-CTGGGCTACACTGAGCACC-3′
Reverse	5′-AAGTGGTCGTTGAGGGCAATG-3′
miR-4656	
Forward	5′- GCCGAGTGGGCTGAGGGCAG-3′
Reverse	5′-CTCAACTGGTGTCGTGGA-3′
U6	
Forward	5′-GTGGACCGCACAAGCTCGCT-3′
Reverse	5′-TTGTTGAACGGCACTGTGTATAGCA-3′

### Western blotting

Total protein lysates were prepared with RIPA lysis buffer (Solarbio, Beijing, China). HDAC11 antibody (58442, 1:2000) was bought from Cell Signaling Technology and β-actin antibody (ab8226, 1:5000) was the product of Abcam. HRP-conjugated secondary antibodies to rabbit (ab6721, 1:10000) and mouse (ab205719, 1:10000) were purchased from Abcam. The proteins were separated by the 8% SDS-PAGE gel and transferred to the PVDF membrane. The membrane was blocked with 5% non-fat milk, incubated with primary antibody and secondary antibody sequentially, then developed with ECL Western Blotting Substrate (Pierce; Thermo Fisher Scientific). HDAC11 was normalized to β-actin to calculate the relative expression with Image J software (V.1.6.1, National Institute of Health).

### Cell proliferation and apoptosis assays

The proliferative ability of cells was measured with a CCK-8 kit (DoJinDo, Shiga, Japan). Briefly, 5000 cells were seeded into each well in the 96-well plate. 0, 24, 48, 72, 96 h post transfection, CCK-8 solution was mixed with culture medium and maintained for 2 h. The absorbance of mixture at 450 nM reflected the cell proliferative ability. For detection of cell apoptosis, flow cytometry analysis was used with an Annexin V-FITC Apoptosis Detection Kit (Merck, Darmstadt, Germany). Briefly, cells were harvested and stained with PI, Annexin V-FITC. Cells were then subjected to flow cytometry analysis. PI +/Annexin V + and PI-/Annexin V + cells were apoptotic cells. The data were analyzed with FlowJo software.

### Nuclear and cytoplasmic extraction

The nuclear and cytoplasmic portion of cells were extracted via a NE-PER^®^ Nuclear and Cytoplasmic Extraction Reagents (Thermo Fisher Scientific) following manufacturer’s protocol. RT-qPCR was performed to detect the relative expression of ST8SIA6-AS1 in cytoplasmic and nuclear fraction of cells. β-actin and U6 were controls for cytoplasmic mRNA and nuclear mRNA respectively.

### Bioinformatic analysis

The RNA sequencing data of HCC and normal tissues from GSE77509 dataset were downloaded and analyzed on the GEO database (https://www.ncbi.nlm.nih.gov/gds/). The expression of ST8SIA6-AS1 in TCGA-LIHC was analyzed on the GEPIA software (http://gepia.cancer-pku.cn/), the software was also used to examine the association between HDAC11 mRNA and ST8SIA6-AS1 expression. The sequence of ST8SIA6-AS1 full length was analyzed in lncLocator software (http://www.csbio.sjtu.edu.cn/bioinf/lncLocator/) to predict the subcellular localization. miRDB software (http://mirdb.org/) was used to predict the complementary binding site of miRNAs in ST8SIA6-AS1. miRDB and TargetScan softwares (http://www.targetscan.org/) were applied to predict target genes of miR-4656.

### Dual luciferase reporter assay

Full length of ST8SIA6-AS1 was subcloned from pcDNA3 into pmirGLO plasmid. 3′UTR of HDAC11 was amplified from Huh7 cDNA and inserted into pmirGLO plasmid. Point mutations were introduced into pmirGLO-ST8SIA6-AS1-WT and pmirGLO-HDAC11 3′UTR-WT with QuickChange Site-Directed Mutagenesis Kit (Stratagene; Agilent Technologies, Santa Clara, CA). The pmirGLO plasmid was co-transfected with miRNA mimic, pcDNA3 plasmid into cells using Lipofectamine 3000 following manufacturer’s guideline. 48 h after transfection, the relative luciferase activity of each group was detected with a Dual Luciferase Reporter Detection System (Promega, Madison, WI).

### Statistical analysis

The data were analyzed with Graphpad Prism 6.0 and presented as mean ± SD. Each experiment was repeated three times. Two groups were compared with Students’ t test. Three groups were compared with one-way ANOVA followed by Tukey’s test. Pearson correlation analysis was used to study the association between expression of two genes in samples. Value of p less than 0.05 was considered to indicate statistically significant.

## Results

### ST8SIA6-AS1 was upregulated in hepatocellular carcinoma (HCC)

**Fig. 1:**
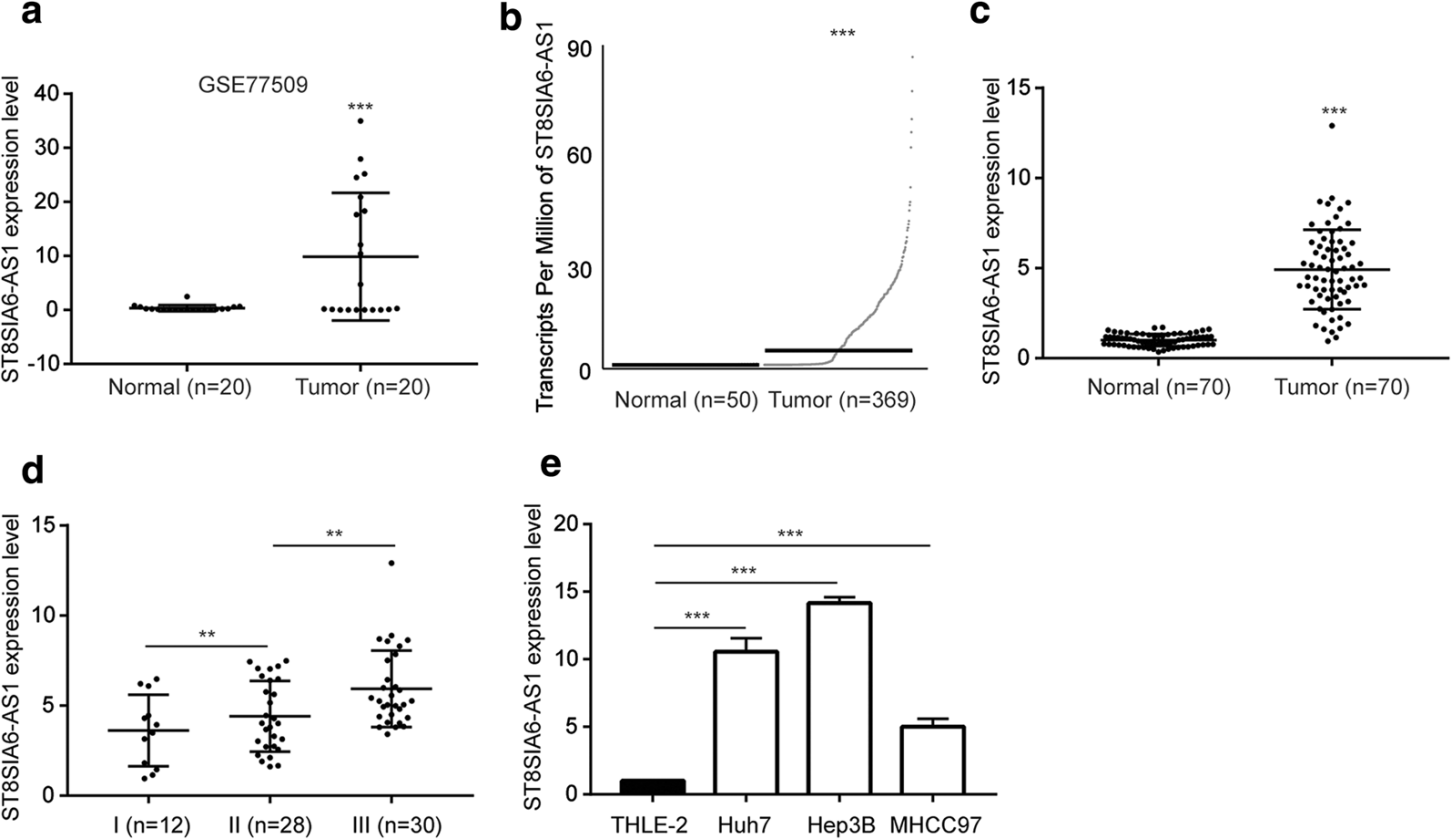
LncRNA ST8SIA6-AS1 was elevated in hepatocellular carcinoma (HCC). **a** Via analyzing previously published RNA sequencing data (GSE77509), lncRNA ST8SIA6-AS1 was found as an upregulated lncRNA in HCC. **b** The TCGA data suggested that ST8SIA6-AS1 was significantly increased in HCC. **c** We collected 70 HCC samples and matched normal samples. RT-qPCR indicated that ST8SIA6-AS1 was increased in HCC samples. **d** ST8SIA6-AS1 was increased in HCC of high-grade in the collected samples. **e** It was found that ST8SIA6-AS1 was increased in HCC cell lines (Huh7, Hep3B, MHCC97) compared with normal liver cells THLE-2. **p < 0.01; ***p < 0.001

A previous RNA-seq study has discovered hundreds of dysregulated lncRNA in HCC compared with normal tissues [17]. We re-analyzed the expression data and found that ST8SIA6-AS1 (ENSG00000204832) was one of most significantly upregulated lncRNAs in 20 HCC samples compared with normal tissues (Fig. 1a). The expression of ST8SIA6-AS1 was further explored in a large cohort from TCGA-LIHC (Liver Hepatocellular Carcinoma) containing data of 369 HCC samples and 50 normal liver samples. Consistently, it was found that ST8SIA6-AS1 was increased in HCC tissues (Fig. 1b). In addition, we collected 70 pairs of HCC samples and matched normal tissues. RT-qPCR results showed that ST8SIA6-AS1 was significantly increased in HCC samples compared to their counterparts (Fig. 1c). Expression of ST8SIA6-AS1 was not associated with sex, age, HBsAG status and tumor size (Table 2). However, highest expression of ST8SIA6-AS1 was observed in tumors of later-stage (Stage III) compared with those of early stage (Stage I and II) (Fig. 1d). Moreover, high expression of ST8SIA6-AS1 was found in a panel of HCC cell lines (Huh7, Hep3B, MHCC97) compared with the immortalized liver cell THLE-2 (Fig. 1e). These data collectively demonstrated that ST8SIA6-AS1 was an upregulated lncRNA in HCC.

**Table 2:** Association of ST8SIA6-AS1 expression with clinicopathological characteristics in 70 patients with hepatocellular carcinoma

Clinicopathological parameters	Relative expression of ST8SIA6-AS1		P value
	High (n = 35)	Low (n = 35)	
Gender			0.465
Male	23	19	
Female	12	16	
Age (years)			0.297
≥ 55	22	27	
< 55	13	8	
Tumor size (cm)			0.223
≥ 5	17	11	
< 5	18	24	
HBsAg			0.611
Positive	22	25	
Negative	13	10	

### Knockdown of ST8SIA6-AS1 inhibited cell proliferation and induced cell apoptosis

**Fig. 2:**
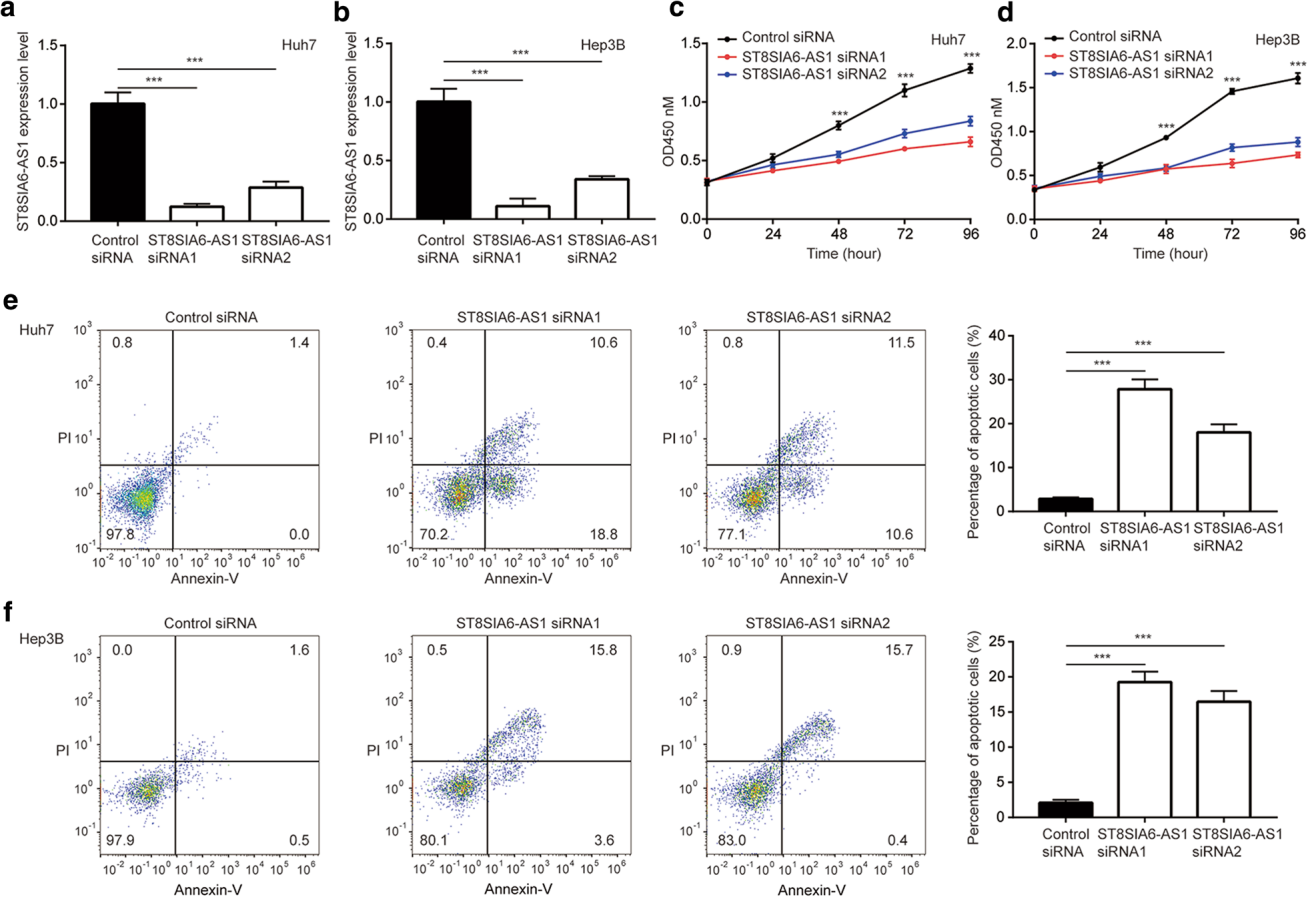
Knockdown of ST8SIA6-AS1 inhibited cell proliferation and evoked cell apoptosis. **a, b** Two ST8SIA6-AS1 siRNAs reduced ST8SIA6-AS1 expression in Huh7 (**a**) and Hep3B (**b**) cells. **c, d** Knockdown of ST8SIA6-AS1 repressed cell proliferation in Huh7 (**c**) and Hep3B (**d**) cells. **e, f** Knockdown of ST8SIA6-AS1 evoked cell apoptosis in Huh7 (**e**) and Hep3B (**f**) cells. ***p < 0.001

We applied siRNA-mediated silencing of ST8SIA6-AS1 expression to study the biological function of ST8SIA6-AS1 in HCC. RT-qPCR verified that transfection of ST8SIA6-AS1 siRNA 1 or ST8SIA6-AS1 siRNA 2 could significantly repressed ST8SIA6-AS1 expression in two HCC cell lines Huh7 and Hep3B (Fig. 2a, b). In the CCK-8 assay, we observed that transfection of either ST8SIA6-AS1 siRNA 1 or ST8SIA6-AS1-2 would reduce the absorbance at 450 nM in Huh7 cells, indicating the proliferation of cells was inhibited (Fig. 2c). Similar results were observed in Hep3B cells (Fig. 2d). With the flow cytometry, it was found that knockdown of ST8SIA6-AS1 elevated percentage of apoptotic cells of Huh7 and Hep3B (Fig. 2e, f). Our data manifested that ST8SIA6-AS1 promoted cell proliferation and inhibited cell apoptosis in HCC.

### ST8SIA6-AS1 was associated with miR-4656 expression in HCC

**Fig. 3:**
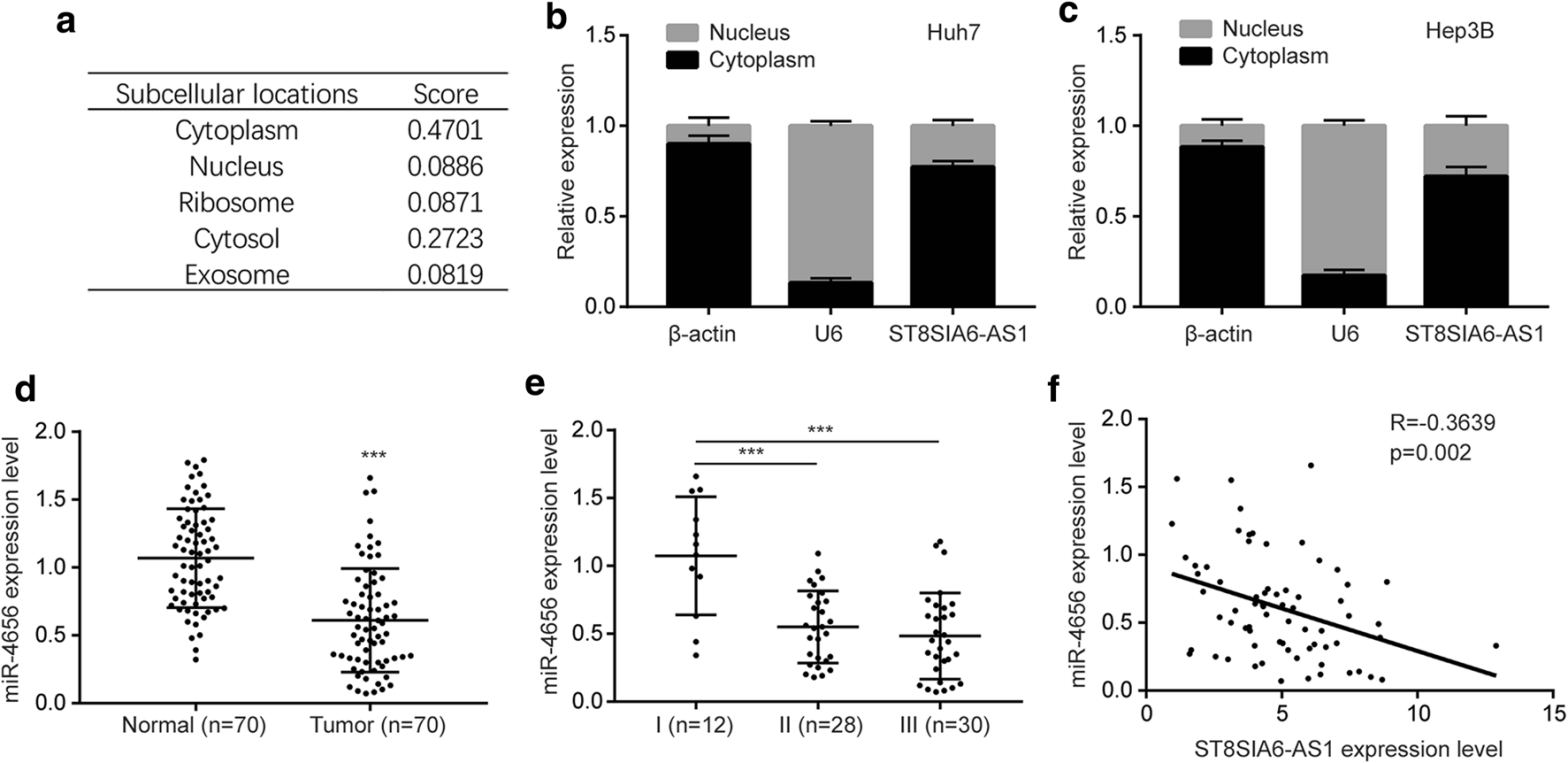
ST8SIA6-AS1 was co-expressed with miR-4656 in HCC. **a** Prediction of ST8SIA6-AS1 subcellular localization was performed on the lncLocator software. **b, c** Cytoplasm and nuclear RNA was separated and RT-qPCR was carried out to detect ST8SIA6-AS1 in cytoplasm and nuclear of Huh7 (**b**) and Hep3B (**c**) cells. **d** RT-qPCR indicated that miR-4656 was decreased in HCC samples compared with matched normal tissues. **e** Relatively lower expression of miR-4656 was observed in high-grade HCC. **f** Pearson correlation analysis manifested a negative correlation between miR-4656 and ST8SIA6-AS1 in HCC. ***p < 0.001

To investigate the molecular mechanisms of ST8SIA6-AS1, we used lncLocator, a bioinformatic tool, to predict the subcellular localization of ST8SIA6-AS1. The result suggested that ST8SIA6-AS1 was mainly located in cytoplasm (Fig. 3a). For validation, we separated cytoplasm and nuclear portion of Huh7 and Hep3B cells. RT-qPCR confirmed that more than 70% of ST8SIA6-AS1 was located in cytoplasm (Fig. 3b, c), indicating ST8SIA6-AS1 might function via the competing endogenous RNA (ceRNA) method. Prediction by miRDB software showed that there were complementary binding sites for several miRNAs in ST8SIA6-AS1. Among them, miR-4656 was a previous reported tumor suppressor in several cancer types [18–20]. To study the expression of miR-4656 in HCC, we performed RT-qPCR in 70 pairs of collected samples. It was revealed that miR-4656 was decreased in HCC samples compared with normal tissues (Fig. 3d). In contrast to ST8SIA6-AS1, miR-4656 was lowly expressed in tumors of later-stage (Stage III) compared with those of early stage (Stage I) (Fig. 3e). Interestingly, the Pearson correlation analysis showed a strong negative correlation between miR-4656 and ST8SIA6-AS1 expression in HCC samples (Fig. 3f), suggesting their regulatory association in HCC.

### ST8SIA6-AS1 sponged miR-4656 in HCC

**Fig. 4:**
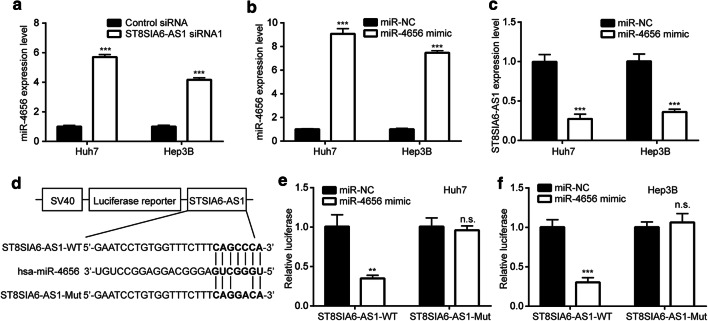
ST8SIA6-AS1 repressed miR-4656 in HCC. **a** Knockdown of ST8SIA6-AS1 increased miR-4656 level in Huh7 and Hep3B cells. **b** miR-4656 mimic increased miR-4656 level in Huh7 and Hep3B cells. **c** miR-4656 decreased ST8SIA6-AS1 level in Huh7 and Hep3B cells. **d** ST8SIA6-AS1-WT (wild type) and ST8SIA6-AS1-Mut (mutant) was inserted into luciferase plasmid. **e, f** Dual luciferase reporter assay confirmed that miR-4656 mimic repressed luciferase activity of ST8SIA6-AS1-WT in Huh7 (**e**) and Hep3B (**f**) cells. **p < 0.01; ***p < 0.001

In Huh7 and Hep3B, knockdown of ST8SIA6-AS1 increased miR-4656 expression (Fig. 4a), indicating ST8SIA6-AS1 might interact with miR-4656 to decrease its expression. To study the impact of miR-4656 on ST8SIA6-AS1, we transfected miR-4656 mimic into HCC cells. As detected by RT-qPCR, miR-4656 mimic increased miR-4656 expression in Huh7 and Hep3B cells (Fig. 4b). Overexpression of miR-4656 decreased ST8SIA6-AS1 expression in Huh7 and Hep3B cells (Fig. 4c). We next constructed luciferase reporter plasmid containing ST8SIA6-AS1 full length (ST8SIA6-AS1-WT) or the mutant form ST8SIA6-AS1 with two-point mutation in the predicted binding site (ST8SIA6-AS1-Mut) (Fig. 4d). In the dual luciferase reporter assay, miR-4656 mimic reduced relative luciferase activity of ST8SIA6-AS1-WT without affecting the luciferase activity of ST8SIA6-AS1-Mut in Huh7 cells (Fig. 4e). The similar results were observed in Hep3B (Fig. 4f).

### ST8SIA6-AS1 upregulated HDAC11 via sponging miR-4656

**Fig. 5:**
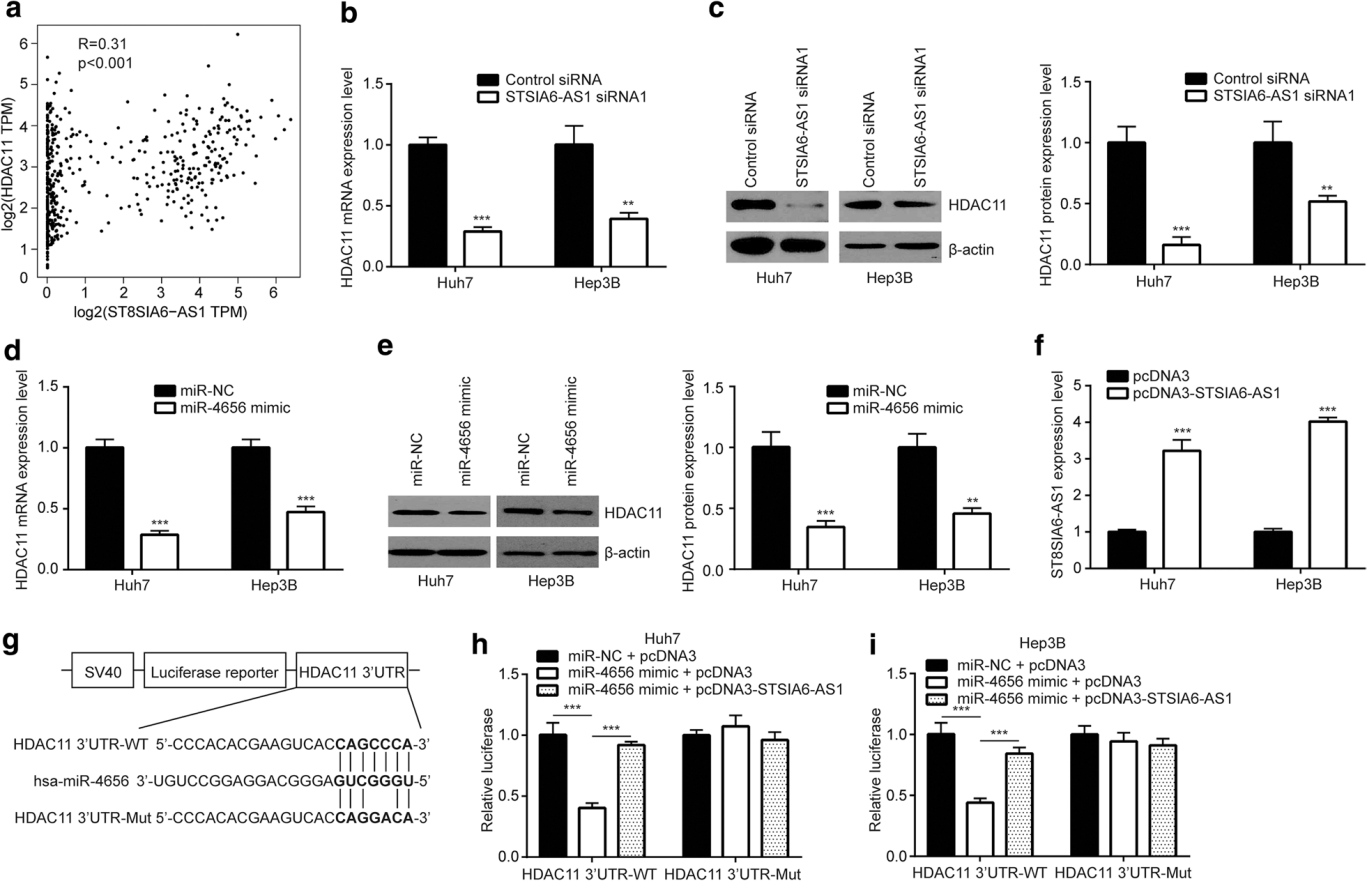
ST8SIA6-AS1 upregulated HDAC11 via sponging miR-4656. **a** Analysis of TCGA data suggested that ST8SIA6-AS1 was positively correlated with HDAC11 expression in HCC. **b** Knockdown of ST8SIA6-AS1 decreased HDAC11 mRNA level in Huh7 and Hep3B cells. **c** Knockdown of ST8SIA6-AS1 decreased HDAC11 protein level in Huh7 and Hep3B cells. **d** miR-4656 mimic decreased HDAC11 mRNA level in Huh7 and Hep3B cells. **e** miR-4656 mimic decreased HDAC11 protein level in Huh7 and Hep3B cells. **f** pcDNA3-ST8SIA6-AS1 increased ST8SIA6-AS1 expression in Huh7 and Hep3B cells. **g** HDAC11 3′UTR-WT (wild type) and HDAC11 3′UTR-Mut (mutant) was inserted into luciferase plasmid. **h, i** Luciferase reporter assay indicated that miR-4656 mimic repressed luciferase activity of HDAC11 3′UTR-WT which was reversed by transfection of pcDNA3-ST8SIA6-AS1 in Huh7 (**h**) and Hep3B (**i**) cells. **p < 0.01; ***p < 0.001

To explore the target genes of miR-4656, we used Targetscan and miRDB softwares. In the list of potential targets, HDAC11 was a highly ranked candidate which was involved in cell proliferation and apoptosis of HCC [16]. We analyzed the expression of ST8SIA6-AS1 and HDAC11 in TCGA-LIHC dataset and found a strong positive correlation between them (Fig. 5a). ST8SIA6-AS1 knockdown decreased mRNA expression of HDAC11 in Huh7 and Hep3B cells (Fig. 5b). Western blotting further showed that the HDAC11 protein expression was decreased upon ST8SIA6-AS1 knockdown (Fig. 5c). Consistent to ST8SIA6-AS1 knockdown, miR-4656 mimic also decreased mRNA expression of HDAC11 in Huh7 and Hep3B cells (Fig. 5d), and western blotting confirmed the downregulation of HDAC11 protein (Fig. 5e). We transfected recombinant ST8SIA6-AS1 to elevate ST8SIA6-AS1 expression in Huh7 and Hep3B cells (Fig. 5f). Luciferase plasmids containing HDAC11 3′UTR or mutant form with two-point mutation in the predicted binding site were constructed (Fig. 5g). In the dual luciferase reporter assay, miR-4656 mimic reduced relative luciferase activity of HDAC11 3′UTR-WT without affecting the luciferase activity of HDAC11 3′UTR-Mut in Huh7 cells (Fig. 5h). The effect of miR-4656 mimic was reversed upon ST8SIA6-AS1 overexpression (Fig. 5h). The similar results were observed in Hep3B (Fig. 5i). These results indicated that ST8SIA6-AS1 promoted HDAC11 expression via repression of miR-4656 in HCC cells.

### ST8SIA6-AS1 regulated cell proliferation and apoptosis via HDAC11 in HCC

**Fig. 6:**
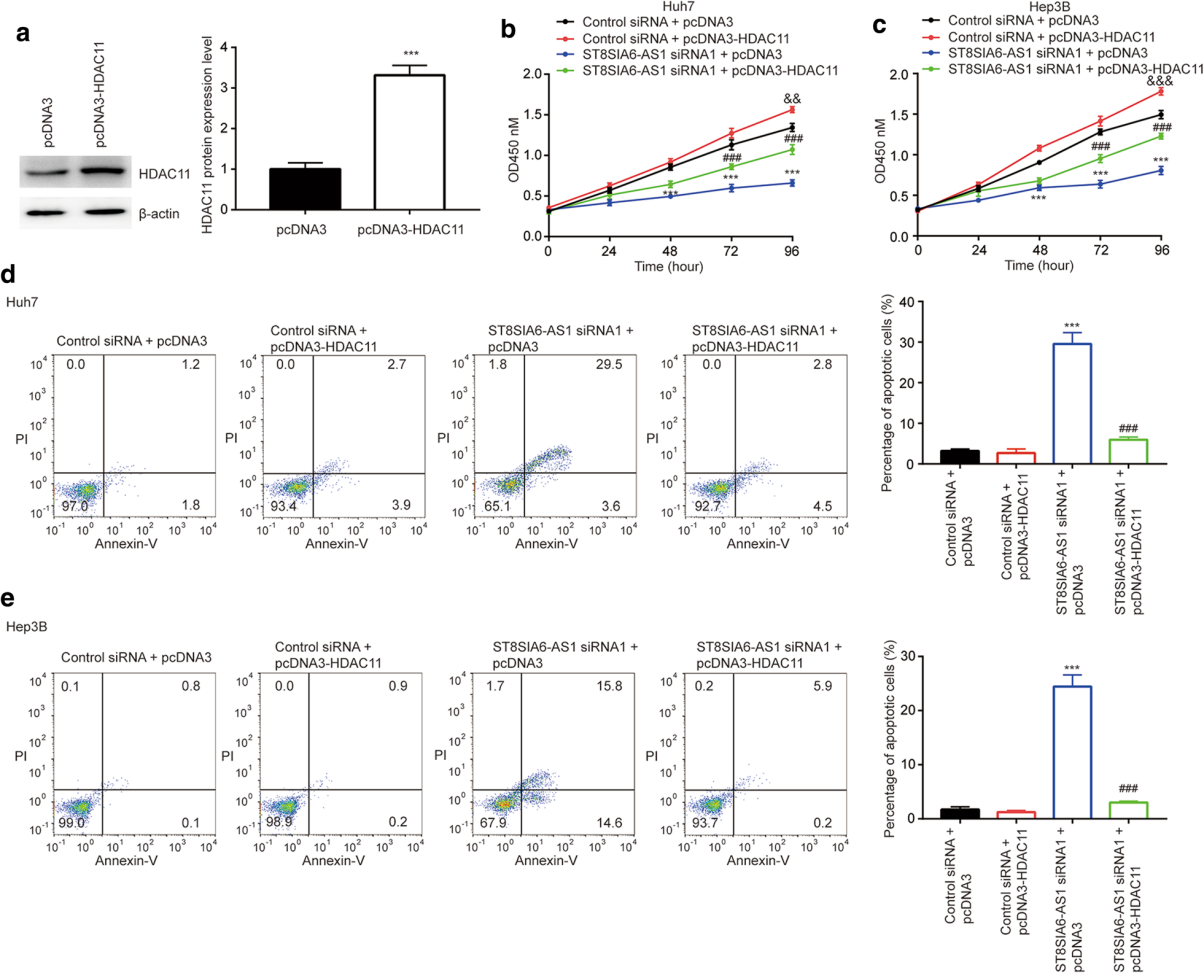
HDAC11 was involved in biological function of ST8SIA6-AS1 in HCC. **a** pcDNA3-HDAC11 increased HDAC11 protein expression in Huh7 cells. **b**, **c** Overexpression of HDAC11 slightly increased cell proliferation and attenuated the anti-proliferation effect of ST8SIA6-AS1 siRNA in Huh7 (**b**) and Hep3B (**c**) cells. **d, e** Overexpression of HDAC11 did not affect cell apoptosis but attenuated the pro-apoptotic effect of ST8SIA6-AS1 siRNA in Huh7 (**d**) and Hep3B (**e**) cells. ***vs. control siRNA + pcDNA3, p < 0.001; ## vs. ST8SIA6-AS1 siRNA + pcDNA3, p < 0.01; ### vs. ST8SIA6-AS1 siRNA + pcDNA3, p < 0.001; && vs. control siRNA + pcDNA3, p < 0.01; &&& vs. control siRNA + pcDNA3, p < 0.001

To investigate the involvement of HDAC11 in the biological function of HDAC11, we constructed plasmid containing full length of HDAC11. Transfection of pcDNA3-HDAC11 increased HDAC11 protein expression in Huh7 cells (Fig. 6a). In the CCK-8 assay, overexpression of HDAC11 slightly increased cell proliferative rate and partially reversed the growth inhibitory effect of ST8SIA6-AS1 in Huh7 and Hep3B cells (Fig. 6b, c). Similarly, with the flow cytometry, overexpression of HDAC11 partially reversed the pro-apoptotic effect of ST8SIA6-AS1 in Huh7 and Hep3B cells, solely elevation of HDAC11 showed little effect on cell apoptosis (Fig. 6d, e). The data showed that HDAC11 was pivotal for the function of ST8SIA6-AS1 in HCC.

## Discussion

Dysregulation of lncRNAs resulted in HCC progression via sustained activation of pro-proliferative signaling and inactivation of pro-apoptotic pathways [8]. Recent studies revealed that upregulation of lncRNAs such as ANRIL, HANR would lead to hepatocellular carcinogenesis by promoting uncontrolled cell proliferation [21, 22]. Luo et al. found ST8SIA6-AS1 accelerated cell cycle progression, proliferation and resistance to cell apoptosis in breast, lung and pancreatic cancer cells [23]. Most recently, it was found that ST8SIA6-AS1 promoted cell proliferation, migration and invasion of breast cancer cells [24]. All these studies demonstrated an oncogenic role of ST8SIA6-AS1 in cancer cells. Via re-analyzing previous study [17], we found ST8SIA6-AS1 was an upregulated lncRNA in HCC. We confirmed the upregulation of ST8SIA6-AS1 in HCC by RT-qPCR. Furthermore, we observed that siRNA-mediated knockdown of ST8SIA6-AS1 repressed HCC cell proliferation and induced cell apoptosis. Thus, ST8SIA6-AS1 was also a lncRNA with oncogenic potential in HCC. In the future study, we will further investigate the potential involvement of ST8SIA6-AS1 in HCC cell metastasis and drug resistance.

Co-expression analysis revealed a lncRNA-associated ceRNA network in HCC with high complexity [25]. Accumulating evidences demonstrated that these upregulated lncRNA exerted their function via sponging tumor suppressive miRNAs [26]. The miRNA sponged by ST8SIA6-AS1 has not been studied yet. We found ST8SIA6-AS1 was mainly located in cytoplasm, indicating ST8SIA6-AS1 might be a regulator of miRNAs in HCC. Among the predicted miRNAs, miR-4656 was associated with cancer progression. In breast cancer, miR-4656 acted downstream of linc00339 and was involved in cancer cell proliferation [18]. MiR-4656 was decreased in prostate cancer and inhibited cell proliferation of cancer cells [19]. It is not known whether miR-4656 was differentially expressed in HCC. We found that miR-4656 was downregulated in HCC which further implied the association between miR-4656 and ST8SIA6-AS1. Our data confirmed that ST8SIA6-AS1 sponged miR-4656 in HCC cells. The study firstly showed that miR-4656 was a downregulated miRNA in HCC and identified ST8SIA6-AS1 as a new regulator of miR-4656. We believe that miR-4656 is crucial for HCC development, which needs further investigation.

HDAC11 was the only member of HDACs pivotal for HCC progression [16]. HDAC11 facilitated metabolism of HCC cells and downregulation of HDAC11 induced cell apoptosis [16, 27]. Although HDAC11 was related to several miRNAs [28, 29], it remains unknown how HDAC11 was regulated by non-coding RNA such as miRNA and lncRNA. We showed that HDAC11 was a target of miR-4656, and ST8SIA6-AS1 promoted HDAC11 expression via sponging miR-4656. Moreover, HDAC11 overexpression attenuated the effect of ST8SIA6-AS1 siRNA in HCC cells. The data supported a pivotal role of the ST8SIA6-AS1/miR-4656/HDAC11 axis in HCC development. However, whether the ST8SIA6-AS1/miR-4656/HDAC11 axis contributes to HCC progression needs further investigation by in vivo models.

## Conclusion

In conclusion, the current study demonstrated a ST8SIA6-AS1/miR-4656/HDAC11 axis in HCC and indicated ST8SIA6-AS1 was an upregulated lncRNA in HCC. Hence, ST8SIA6-AS1 was a promising biomarker for patients with HCC.

## Data Availability

All data generated or analyzed during this study are included in this published article.
